# Overranging and overbeaming measurement in area detector computed tomography: A method for simultaneous measurement in volume helical acquisition

**DOI:** 10.1002/acm2.12650

**Published:** 2019-06-04

**Authors:** Atsushi Urikura, Takanori Hara, Tsukasa Yoshida, Eiji Nishimaru, Takashi Hoshino, Yoshihiro Nakaya, Masahiro Endo

**Affiliations:** ^1^ Division of Diagnostic Radiology Shizuoka Cancer Center Nagaizumi Japan; ^2^ Department of Medical Technology Nakatsugawa Municipal General Hospital Nakatsugawa Japan; ^3^ Radiation and Proton Therapy Center Shizuoka Cancer Center Nagaizumi Japan; ^4^ Department of Radiology Hiroshima University Hospital Hiroshima Japan; ^5^ Department of Radiological technology Osaka College of High Technology Osaka Japan

**Keywords:** computed tomography, dose length product, dosimetry, overbeaming, overranging

## Abstract

**Purpose:**

We propose a novel method to assess overbeaming and overranging, as well as the effect of reducing longitudinal exposure range, by using a dynamic z‐collimator in area detector computed tomography.

**Methods and materials:**

A 500‐mm diameter cylindrical imaging plate was exposed by helical scanning in a dark room. The beam collimation of the helical acquisitions was set at 32 and 80 mm. Overbeaming and overranging with the dynamic z‐collimator were measured.

**Results:**

The actual beam widths were approximately 39 and 88 mm at 32 and 80 mm collimation, respectively, and were relatively reduced owing to increased beam collimation. Overranging was 27.0 and 48.2 mm with a pitch of 0.83 and 1.49 at 32 mm collimation and 72.5 and 83.1 mm with a pitch of 0.87 and 0.99 at 80 mm collimation. The dynamic z‐collimator relatively reduced the overranging by 17.3% and 17.1% for the 32 and 80 mm collimation, respectively.

**Conclusion:**

We devised a method to simultaneously measure overbeaming and overranging with only one helical acquisition. Although the dynamic z‐collimator reduced the overranging by approximately 17%, wider collimation widths and higher pitch settings would increase the exposure dose outside the scan range.

## 1 INTRODUCTION

Advances in multirow detector computed tomography (MDCT) have made high‐speed scanning an easy operation to perform. In area detector computed tomography (CT), volume helical scanning can be performed with a beam width (BW) set up to 80 mm in the *z*‐direction with current scanners.^1^ To avoid radiosensitive organs while attaining diagnostic objectives, the operator sets the scan length with millimeter accuracy at the scanner console. However, the longitudinal x‐ray fluence of MDCT contains the penumbra, called overbeaming, for homogenizing the x‐ray intensity incident on the detector.^2^ Considering that the influence of overbeaming in MDCT varies depending on the number of detector configurations, BW, and scanning geometry, it is scanner specific. Although overbeaming relatively decreases owing to the increased detector row,^3^, ^4^ overbeaming in recent volume helical scanning has not necessarily been clarified.

X‐ray exposure in helical scanning is extended outside of the set scan range along the *z*‐direction. The exposure length extension of helical scanning, which is called overranging, tends to increase because of the larger detector coverage and pitch selection.^5^, ^6^, ^7^ Increased overranging in helical scanning could not be ignored in children with a short scan range or in the case of radiosensitive organs located near the scan range.^8^ Radiation exposure to radiosensitive organs outside the scan range extending in the *z*‐direction should be minimized. To reduce the increased overranging in MDCT, a dynamic *z*‐collimator was installed in advanced CT scanners.^5^, ^9^, ^10^ Shirasaka et al.^11^ measured overranging in a 128‐detector row CT scanner (Brilliance iCT; Philips Healthcare, Cleveland, OH, USA) and concluded that the spiral dynamic *z*‐collimator is important for unnecessary overrange dose reduction.

Measurement methods have already been established for overranging and overbeaming. Generally, individual measurements are performed to quantify overranging and overbeaming. A film method is used to measure overbeaming, which is evaluated using the full width at half maximum (FWHM) of the dose profile.^12^ A computed radiography imaging plate data has been reported to be used for the measurement of CT collimation width, including overbeaming for quality control scripts.^13^ To measure overranging, a method using a film or dosimeter is performed.^5^, ^6^ Although the measurement of overranging and overbeaming for volume helical scanning is important for exposure dose management of patients, measuring overranging and overbeaming with several scan parameters is laborious. We proposed a novel method to quantify overranging and overbeaming by using only one helical acquisition. To our knowledge, no report has evaluated overranging and overbeaming measurements by using one helical acquisition. We proposed a novel method to assess overbeaming and overranging, as well as the effect of reducing longitudinal exposure range, by using a dynamic z‐collimator.

## 2 METHODS

### 2.A Measurement procedure and helical acquisition parameters

**Figure 1:**
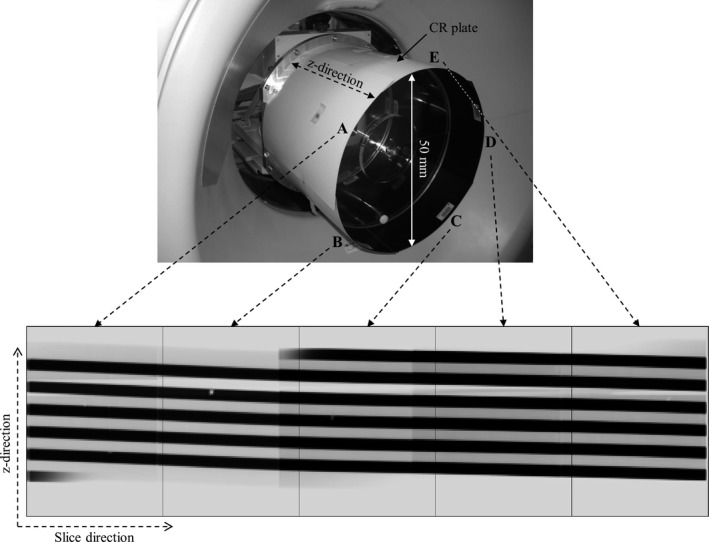
Five imaging plates (a–e) are cylindrically placed around a 500‐mm diameter acrylic phantom and at the center of the rotation. A stripe image obtained by developing scanned cylindrical images. The black stripes indicate the scan orbit corresponding to the helical acquisition

In our proposed measurement method, five imaging plates for computed radiography (35 cm × 43 cm ST‐V CR plates; Fujifilm Medical, Tokyo, Japan) were placed cylindrically around an acrylic phantom (500 mm diameter; Fig. 1) and then exposed by helical scanning in a darkroom. Thereafter, the five CR plates were loaded into Fuji imaging plate cassettes and processed in an XG‐1 (Fujifilm Medical) reader. The images were saved in Digital Imaging and Communications (DICOM) in Medicine format. The five scanned CR plates were developed and synthesized as one image using ImageJ software (ver. 1.49d; National Institutes of Health, Bethesda, MD, USA), and a stripe image was acquired. This stripe image provided the trajectory of the x‐ray fluence scanned on a cylindrical‐shaped CR plate as a development image. The pixel value (PV) of the striped image was converted into an effective exposure (*E*) using the following equation in a manner similar to the previous work for the digital radiography system^14^:

(1) $$E=10^{\text{PV}/G}$$

where *G* is the gray level, and the *G* of this CR system was 1024.

All scan acquisitions were performed with a 320‐row area detector CT scanner (Aquilion ONE; Canon Medical Systems, Tokyo, Japan) by using conventional (32 mm width) and volume (80 mm width) helical scanning. The pitch was set at 0.83 and 1.49 for 32 mm and at 0.87 and 0.99 for 80‐mm beam collimation, respectively. Furthermore, the overranging reduction by a dynamic z‐collimator (active collimator, Canon Medical Systems) was assessed. The other scan parameters were set at 80 kVp, 10 mA, 0.5 s/rotation, and 160 mm scan range.

### 2.B Overbeaming measurement

**Figure 2:**
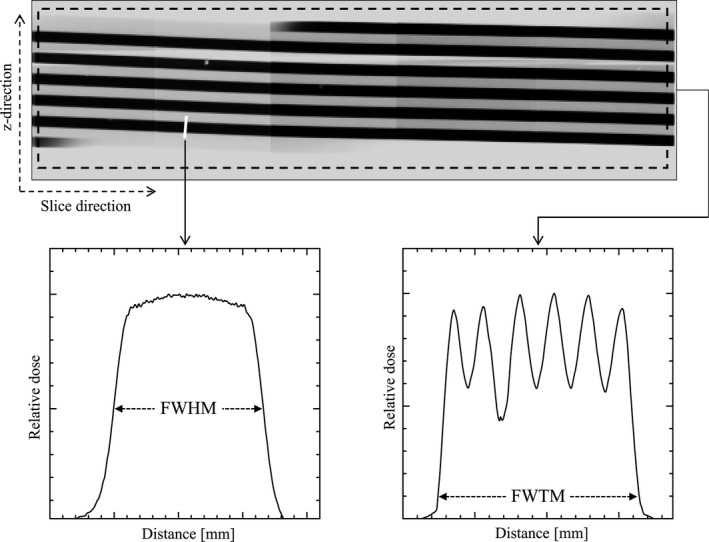
The profile in the perpendicular direction to a single stripe is plotted (white line). For actual beam width measurements, the full width at half maximum was measured (left bottom graph). The dash line depicts the placement of the region of interest to measure overranging. The mean values along the slice direction were acquired, and profiles along the z‐direction were plotted (right bottom graph)

For overbeaming measurements, the profile in the perpendicular direction to a single stripe was plotted (bottom left graph, Fig. 2). We obtained three perpendicular profiles from arbitrary stripes in each stripe image. The tilt angle of the stripe was determined using ImageJ software. The actual BW at the rotation center was inverse‐square corrected using the measured FWHM of the profile curve and was calculated using the following equation:

(2) $$\text{BW}\left(\text{mm}\right)=\text{FWHM}\times \text{FID}/\text{FSD}$$

where FWHM is the measured FWHM at the stripe image, and FSD and FID are the respective focus surface and focus isocenter distances corresponding to the geometric arrangement of the CT scanner.^15^ The actual *BW* was the estimated actual BW at the rotation center. The dose efficiency (DE)^2^ was calculated by dividing the nominal BW by the actual BW.

### 2.C Overranging measurement

To measure overranging, a rectangular region of interest (ROI) containing the entire stripe image was placed (dashed line, Fig. 2). The mean values along the slice direction were acquired, and profiles along the *z*‐direction were plotted (bottom right graph, Fig. 2). The overranging was calculated by the following equation:

(3) Overranging=(FWTM-d)/2

where FWTM is the measured full width at tenth maximum at the profile, and *d* is the scan range. The FWTM of the profile was defined as the actual exposure length along the *z*‐direction.

Furthermore, overranging was compared with and without the use of an active collimation at the same scan parameters. The relative reduction rate (%) of overranging with and without an active collimation was measured, and the dose length products (mGy cm) displayed at the CT console were recorded. With and without active collimation measurements, a high‐pitch setting (1.49 and 0.99) was used for 32 and 80 mm BWs.

### 2.D Accuracy of the stripe image measurement

To verify the accuracy of the calculated overbeaming and overranging, we compared the values measured by our method and a conventional method. In the conventional method,^12^ the CR plate was placed as close as possible to the center of the rotation by adjusting the height of the CT couch. To measure the dose profile for overbeaming and overranging, nonhelical and helical scans were performed, respectively. The scanned CR plate was processed in an XG‐1 reader. Thereafter, the value along the *z*‐direction was plotted. The FWHM of the dose profile was defined as the actual BW. Overranging was measured by performing a helical scan using the placement of the CR plate described above. The FWTM of the profile curve was defined as the actual exposure length along the *z*‐direction, and the overranging was calculated by eq. (3).

## 3 RESULTS

Table 1 shows the results of overbeaming and DE with respective beam collimations and pitch settings. BWs were approximately 39 and 88 mm at 32 and 80 mm collimation, respectively. The differences between the conventional method and our method were maximums of 1.0 and 4.7 mm for 32 and 80 mm collimation, respectively, and the DEs for the conventional method and our method were 0.82–0.84 and 0.90–0.95, respectively.

**Table 1:** Overbeaming and dose efficiency results obtained by various beam collimations and pitches.

Beam collimation (mm)	Pitch	Beam width (mm)	Dose efficiency
32	Conv.	39.0 ± 0.10	0.82 ± 0.00
	0.83	38.8 ± 0.74	0.82 ± 0.01
	1.48	38.0 ± 0.54	0.84 ± 0.01
80	Conv.	83.7 ± 0.10	0.95 ± 0.01
	0.87	87.8 ± 0.50	0.91 ± 0.01
	0.99	88.4 ± 0.52	0.90 ± 0.01

Data are represented as mean ± SD.

Conv, Measurement by conventional method.

**Figure 3:**
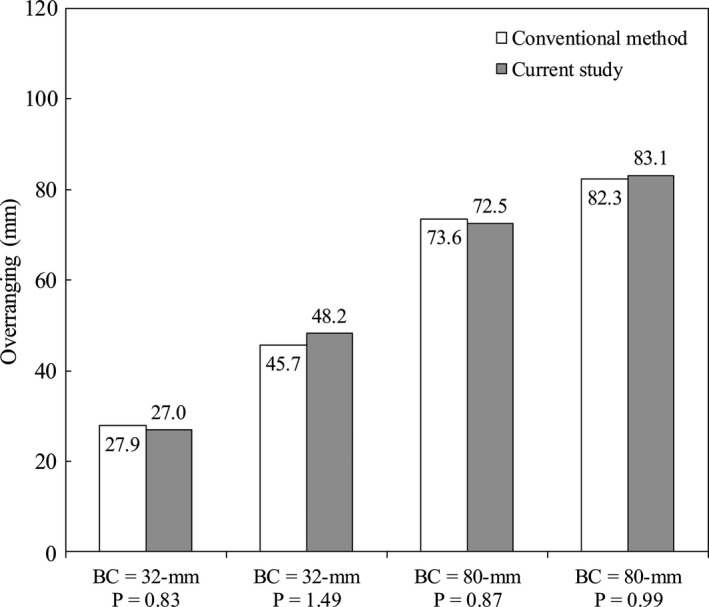
Graph showing overranging for various beam collimations (BC) and pitch (P). Overranging increased corresponding to the P and BC. The maximum overranging values were 48.2 and 83.1 mm for 32 and 80 mm BCs, respectively.

Figure 3 shows the results of overranging with respective beam collimations and pitch settings. Overranging increased corresponding to the pitch and nominal beam collimation. Overranging showed the smallest value at 32 mm collimation (pitch = 0.83) and the largest value at 80 mm (pitch = 0.99).

Table 2 shows the results for overranging with and without active collimation. Overranging showed the largest (100.3 mm) at 80 mm collimation without active collimation. Relative overranging reduction by active collimation was 17.3% and 17.1% at 32 and 80 mm collimation, respectively.

**Table 2:** Results for overranging with/without the active collimator.

Beam collimation (mm)	Active collimator	DLP (mGy cm)	Actual exposure length (mm)	Overranging (mm)	Overranging reduction ratio (%)
32	w.o. AC	247.3	276.6	58.3	
	w. AC	233.0	256.3	48.2	17.3
80	w.o. AC	401.5	360.6	100.3	
	w. AC	385.1	326.2	83.1	17.1

AC, active collimator; DLP, dose length product.

**Figure 4:**
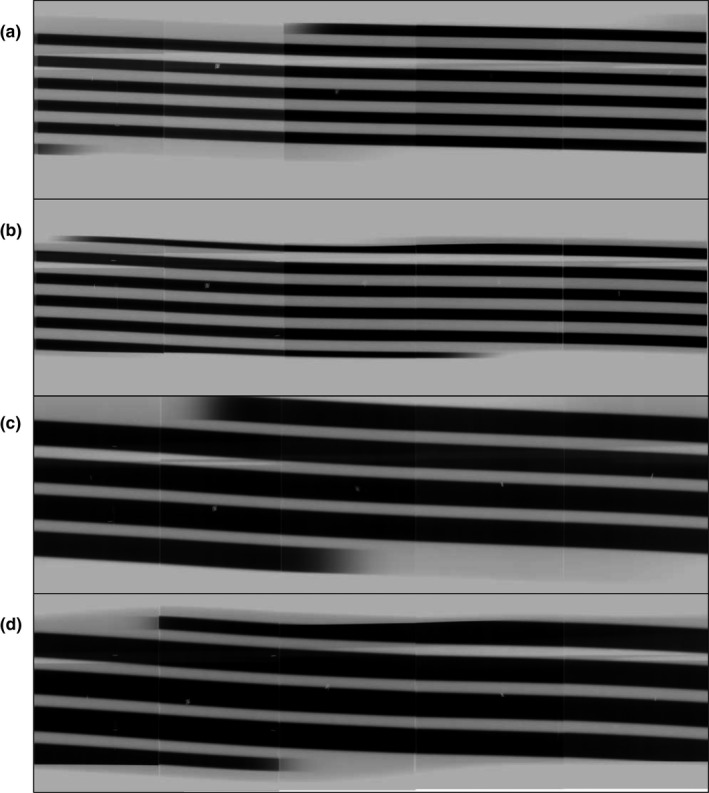
Stripe images acquired without/with an active collimator. The beam collimation was set at 32 (a, b) and 80 mm (c, d). The stripe image depicts that the shade is tapered at the beginning and end according to the action of the active collimator.

Figure 4 shows the stripe images obtained with and without active collimation for 32‐ and 80‐mm beam collimations. The stripe image depicts whether the shade is tapered at the beginning and end according to the action of active collimation.

## 4 DISCUSSION

To our knowledge, this is the first study that documents a method for simultaneously measuring overbeaming and overranging with only one helical scanning. The result demonstrated that the current method was not only effective for accurate measurement but was also easy to measure. Previous studies using x‐ray films or CR plates have also been reported. Tsalafoutas et al.^6^ measured overranging by using three different MDCT scanners and reported detailed results demonstrating that the measuring method of overranging using x‐ray film and CR plates was simple and had high precision. However, simultaneously assessing the overbeaming, overranging, and characterization of the dynamic z‐collimator was difficult.

The result of overbeaming was relatively reduced owing to the increased BW. Considering that overbeaming is caused by the width of the penumbra of the irradiated x‐ray fluence along the *z*‐direction, DE depends on the number of rows (beam collimation). Our results revealed that DE improves in volume helical scanning (≥64 row detector configuration).

**Figure 5:**
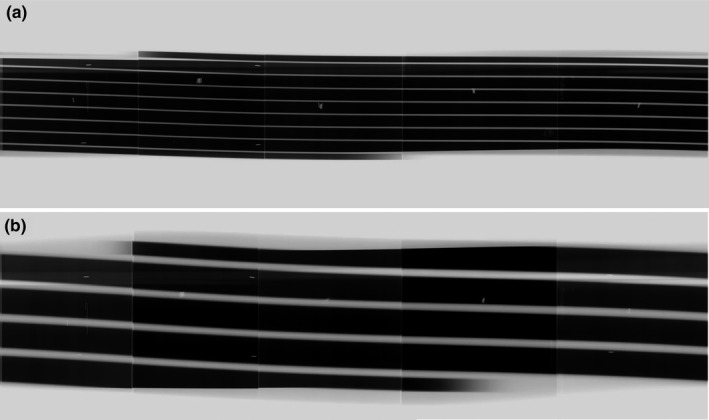
Stripe images acquired by the pitch with < 1.0 (a: 32‐mm beam collimation with pitch of 0.83; b: 80‐mm beam collimation with pitch of 0.87). The large diameter of the measuring instrument made it possible to separately display each stripe even at a low pitch.

In our study the spiral activation of the x‐ray fluence must be irradiated as an independent stripe to measure overbeaming. At a pitch of ≤ 1.0, the stripes overlapped each other at the rotation center. To solve this problem, we used a large diameter measuring instrument, thus making it possible to display each stripe separately even at a low pitch (Fig. 5). The differences in measured BWs at two different pitches were 0.4 and 0.2 mm at 32 and 80 mm of the nominal beam collimations, respectively, thus indicating that this method is highly reproducible. Furthermore, the measurement of overbeaming is generally performed with a fixed x‐ray tube position or with nonhelical acquisitions. To our knowledge, no report has evaluated overbeaming measurement by using helical acquisition.

Overranging was increased owing to the beam collimation and pitch. Understandably, overranging occurred at the beginning and end of the scan range of the helical acquisition. Therefore, despite the short scan range, scanning with the wide BW and high‐pitch setting relatively increased exposure outside the scan range. In our study, overranging reached approximately 83 mm at a BW of 80 mm and a pitch of 0.994. If some radiosensitive organs (eye lens, thyroid gland, mammary gland, etc.) or implantable electric devices (pacemaker, implantable cardioverter–defibrillator, etc.) are near the scan range, operators should pay particular attention to the scan parameters.^16^, ^17^, ^18^, ^19^, ^20^ Mosher et al.^18^ objectively evaluated the exposure dose of neck CT by using a computer simulation. We aimed to reduce the exposure dose of radiosensitive organs by changing the position of the neck and scan parameters in neck CT, and the results showed that the overranging greatly influenced the eye‐lens dose.

Our results showed that active collimator is effective for reducing overranging. It is an indispensable mechanism for overranging reduction during volume helical scanning. Shirasaka et al.^11^ measured overranging in a 128‐detector row CT scanner. Brilliance iCT can also perform helical acquisitions of up to 80 mm coverage. To measure the overrange areas, they used radiophotoluminescent glass rod dosimeters. Helical acquisition (80 mm coverage) with the spiral dynamic z‐collimator demonstrated that the dose‐saving ratios for beam pitches of 0.60, 0.80, and 0.99 were 35.07%, 24.76%, and 13.51%, respectively. Our results showed overranging reduction rates of 17.3% and 17.1% at 32‐ and 80‐mm beam collimation, respectively. With an 80‐mm beam collimation, the active collimator showed a better reduction effect than the dynamic z‐collimator of iCT.

The stripe image depicts that the shade is tapered at the beginning and end according to the action of active collimator. The stripe image visually showed that one side of the active collimator operated asymmetrically and that the BW at the scan start and end was approximately half of the set BW. However, the active collimator and the detailed characteristics of the dynamic z‐collimator installed in each manufacturer’s CT scanners are not clarified. The stripe image we devised made it possible to quantitatively and visually acquire detailed information on the asymmetric operation of the collimator operation from start to end.

Our study involved several potential limitations. First, measurements were performed using the CR plates of a computed radiography system. Given that the CR plate can erase the exposure information, it is effective for repeated measurements. However, many radiology departments are getting rid of their CR systems in favor of digital radiography systems. This problem might be solved using radiochromic film^21^ instead of CR plates. Second, our study showed results of limited scan parameter combinations. Other beam collimations, pitches, and rotational times were not evaluated. Third, although the degree of overranging reduction by the active collimation might vary depending on the CT scanner generations, our study did not disclose it. Forth, the accuracy of the repeated measurement of overranging for our method did not disclose it, would be future research subjects.

In conclusion, our novel measurement method allowed us to simultaneously measure overbeaming and overranging with only one helical acquisition. Overbeaming was found to relatively decrease as the BW increased. Although the active collimator reduced overranging by up to 17%, wider beam collimation and higher pitch settings increased the exposure dose outside of the scan range.

## CONFLICT OF INTEREST

No conflict of interest.
